# Prevention and Management of Variceal Hemorrhage

**DOI:** 10.1155/2013/434609

**Published:** 2013-03-31

**Authors:** Dong Hyun Kim, Jun Yong Park

**Affiliations:** ^1^Department of Internal Medicine, Yonsei University College of Medicine, Seoul 120-752, Republic of Korea; ^2^Institute of Gastroenterology, Yonsei University College of Medicine, Seoul 120-752, Republic of Korea; ^3^Liver Cirrhosis Clinical Research Center, Seoul 120-752, Republic of Korea

## Abstract

Variceal hemorrhage is a common and devastating complication of portal hypertension and is a leading cause of death in patients with cirrhosis. The management of gastroesophageal varices has evolved over the last decade resulting in improved mortality and morbidity rates. Regarding the primary prevention of variceal hemorrhaging, nonselective **β**-blockers should be the first-line therapy in all patients with medium to large varices and in patients with small varices associated with high-risk features such as red wale marks and/or advanced cirrhosis. EVL should be offered in cases of intolerance or side effects to **β**-blockers, or for patients at high-risk for variceal bleeding who have medium or large varices with red wale marks or advanced liver cirrhosis. In acute bleeding, vasoactive agents should be initiated along with antibiotics followed by EVL or endoscopic sclerotherapy (if EVL is technically difficult) within the first 12 hours of presentation. Where available, terlipressin is the preferred agent because of its safety profile and it represents the only drug with a proven efficacy in improving survival. All patients surviving an episode of bleeding should undergo further prophylaxis to prevent rebleeding with EVL and nonselective **β**-blockers.

## 1. Introduction

Portal hypertension is the main complication of cirrhosis and the gradient between portal pressure and inferior vena cava pressure, the hepatic venous pressure gradient (HVPG), is increased over the normal value of 5 mmHg. Clinically significant portal hypertension is defined as having an HVPG of 10 mmHg or more. Esophageal varices are present in nearly 30% to 40% of patients with compensated cirrhosis and in 60% of those with decompensated cirrhosis [1]. Variceal hemorrhages occur only when there is a clinically significant portal hypertension, defined as HVPG > 12 mmHg [2]. Variceal hemorrhage is perhaps the most devastating portal hypertension-related complication in patients with cirrhosis, occurring in up to 30% of such individuals during the course of their illness. Moreover, variceal hemorrhage leads to deterioration in liver function and is a common trigger for other complications of cirrhosis, such as bacterial infections or hepatorenal syndrome. The 1-year rate of a first bleeding episode is 5–15% and its risk is defined by variceal size, red signs on the varices, and severity of liver disease in patients [3]. As many as 70% of survivors have recurrent bleeding within 1 year after the index hemorrhage [4]. Although mortality rates of variceal hemorrhage in patients with cirrhosis have been falling over the last few decades due to the implementation of effective treatments and improvements in general medical care, it still carries a mortality rate of up to 20% within 6 weeks of the bleeding episode [5, 6]. Management of patients with gastroesophageal varices includes: prevention of varices (preprimary prophylaxis), primary prophylaxis to prevent the initial bleeding episode, the control of an acute hemorrhage, and the prevention of recurrent bleeding after a first episode (secondary prophylaxis). This review summarizes the current management and prevention of variceal hemorrhage. 

## 2. Prevention of Varices and Primary Prophylaxis to Prevent a First Variceal Hemorrhage 

Every patient with a new diagnosis of cirrhosis should have an esophagogastroduodenoscopy to look for the presence and size of varices. Nonselective *β*-blockers reduce portal pressure by an average of 15–20%. However, in patients without varices, a large multicenter, placebo-controlled, double-blinded trial failed to show any benefits of nonselective *β*-blockers (timolol) in the prevention of varices [7]. Serious adverse events were more common among patients in the timolol group. Therefore, treatment with nonselective *β*-blockers is not recommended in this setting.

 Primary prophylaxis is the prevention of the first variceal bleeding in patients with varices. The most common means of primary prophylaxis are either pharmacological or by endoscopy. 

 The nonselective *β*-blockers have been the most widely studied medications in randomized controlled trials evaluating the efficacy of primary prophylaxis in patients with portal hypertension. In patients with low-risk, small varices (without red wale marks and in the absence of severe liver disease), there is limited evidence that shows that their growth may be slowed by the use of nonselective *β*-blockers [8]. Therefore, patients with small varices not using nonselective *β*-blockers, should be considered for endoscopy every 2 years to evaluate the progression of varices [9]. In patients with small varices that are associated with a high-risk of hemorrhaging (varices with red wale marks or varices in a patient with Child-Pugh B or C disease), nonselective *β*-blockers are recommended [3, 10]. In patients with medium/large varices, a meta-analysis of 11 trials that included 1,189 patients evaluating nonselective *β*-blockers (ex. propranolol and nadolol) versus no active treatment or placebo in the prevention of first variceal hemorrhage showed that *β*-blocker reduced the bleeding risk from 30% to 14% (relative risk reduction of 47%) [11]. The number of patients that needed to be treated (NNT) with *β*-blockers to prevent one bleeding episode was estimated to be 10. Also, mortality was reduced with *β*-blockers. Once a patient is started on a *β*-blocker to prevent variceal hemorrhage, the treatment should be lifelong one because the bleeding risk returns to the baseline if the treatment is withdrawn [12].

The dose of *β*-blockers is titrated on the basis of clinical measurements by incremental increases in dosage to reach an endpoint resting heart rate of 55 beats per minute, a reduction of 25% from the baseline rate, or the development of side effects. The advantages of nonselective *β*-blockers are that their cost is low, expertise is not required for their use, and they may prevent other complications, such as bleeding from portal hypertensive gastropathy, ascites, and spontaneous bacterial peritonitis because they reduce portal pressure [13, 14]. However, the use of *β*-blockers is limited by their side-effect profile, which includes hypotension, fatigue, lethargy, depression, and dyspnea in patients with associated pulmonary disease. Due to concomitant diseases such as reactive airway disease, congestive heart failure, bradycardia, and heart block, 15–20% of patients are unable to take *β*-blockers. 

Carvedilol, a nonselective *β*-blocker with an added vasodilatory effect through intrinsic *α*1-adrenergic activity, has been shown to produce a greater decrease in portal pressure than propranolol, an effect probably related to an associated decrease in hepatic and portocollateral resistance [15]. However, the vasodilating effect also causes mild systemic hypotension, which may be of concern in decompensated cirrhosis. In a recent randomized, controlled trial, it was associated with lower rates of first variceal hemorrhage (10% versus 23%) than endoscopic variceal ligation (EVL) and had an acceptable side effect profile [16]. More data will be needed on its effectiveness and long-term safety. 

The efficacy and safety of losartan and irbesartan, angiotensin-II-receptor blockers, in lowering portal pressure has been established in cirrhosis patients [17], but the risk of systemic hypotension and renal failure precludes their use in patients with decompensated cirrhosis [18, 19]. Currently, angiotensin-II-receptor blockers are not recommended in the setting of portal hypertension. 

Endoscopic therapy is another mean of primary prophylaxis. The success of endoscopic sclerotherapy in the treatment of acute variceal bleeding led to the extensive evaluation of sclerotherapy for the prevention of the first variceal bleeding. While early studies showed promising results [20, 21], subsequent larger trials showed no benefit [22, 23], and a prospective, randomized trial was terminated prematurely because of an increased mortality rate in the endoscopic sclerotherapy group [24]. Based on this data, endoscopic sclerotherapy is not recommended for primary prophylaxis.

In recent years, EVL has replaced endoscopic sclerotherapy. In patients with medium or large varices, either nonselective *β*-blockers or EVL can be used, since a meta-analysis of high-quality, randomized, controlled trials has shown equivalent efficacy and no differences in survival [25]. However, EVL should be preferred for patients at high-risk for variceal bleeding who have medium or large varices with red wale marks or advanced liver cirrhosis. Combination therapy with nonselective *β*-blockers and EVL does not seem to confer any additional benefit because addition of *β*-blockers does not decrease the probability of first bleed or death in patients on EVL, but increased the side effects [26].

## 3. The Management of Acute Hemorrhage

The rate of death from acute variceal hemorrhage has been decreasing over the past two decades, probably as a result of improved general management (with short-term antibiotic prophylaxis) and more effective therapies (EVL and vasoactive drugs) [27]. The management of acute variceal hemorrhage consists of general care, such as adequate fluid resuscitation, airway protection, and prophylactic antibiotics, and specific therapy, such as vasoactive drugs, endoscopic treatment, or surgical or radiological shunts. 

The basic medical principles of airway, breathing and circulation are followed to achieve hemodynamic stability. Airway protection should be provided, especially in patients with hepatic encephalopathy, since the patient is at risk for bronchial aspiration of gastric contents and blood. Tracheal intubation is mandatory if there is any concern about the safety of the airway. Blood volume replacement should be initiated as soon as possible with plasma expanders, aiming at maintaining a systolic blood pressure of approximately 100 mmHg. Avoiding prolonged hypotension is particularly important to prevent infection and renal failure, which are associated with increased risk for rebleeding and death [28]. Packed red blood cells are transfused conservatively to keep the target hemoglobin level between 7 and 8 g/dl, as excessive blood volume replacement can increase portal pressure and the risk of rebleeding [6, 29]. Fresh frozen plasma and platelets, although frequently used, do not reliably correct coagulopathy and can induce volume overload [30]. 

Cirrhosis is frequently associated with defects in both humoral and cellular host defense, hence increasing the risk for infection. The most frequent infections are spontaneous bacterial peritonitis (50%), urinary tract infections (25%), and pneumonia (25%) [31]. Two meta-analyses have shown that prophylactic antibiotics in patients with acute variceal hemorrhage prevent infection and significantly increase the short-term survival rate [32, 33]. Therefore, antibiotic prophylaxis is an essential part of therapy for patients with cirrhosis presenting with upper gastrointestinal bleeding and should be instituted from admission. Antibiotic prophylaxis with ceftriaxone (1 g/day for 7 days) is recommended in patients with severe liver disease, high prevalence of quinolone resistance, or prior quinolone prophylaxis, whereas others can receive oral norfloxacin [34, 35].

In suspected variceal hemorrhages, vasoactive drugs need to be started as soon as possible, prior to diagnostic endoscopy. Vasoactive therapy should be maintained for up to 5 days depending on control of bleeding and severity of liver disease. They improve the control of variceal hemorrhage when combined with endoscopic therapy and when compared to endoscopic therapy alone [36]. Vasoactive treatment aims at controlling the bleeding episode by lowering the portal pressure and decreasing variceal blood flow. Two types of vasoactive drugs that are used currently in the management of acute variceal bleeding are present: vasopressin and its analogs (terlipressin) and somatostatin and its analogs (octreotide/vapreotide). In practice, the choice of drugs is usually based on availability and cost.

Vasopressin, which is a powerful vasoconstrictor, lowers portal pressure but also causes systemic vasoconstriction. But, its use is limited by multiple side-effects related to splanchnic vasoconstriction (e.g., bowel ischemia) and systemic vasoconstriction (e.g., hypertension, myocardial ischemia). Terlipressin is a synthetic vasopressin analog with a prolonged half-life and possessing far fewer side effects. Side effects may include transient abdominal pain as well as self-limited diarrhea. There was a statistically significant reduction in failure to control bleeding with terlipressin compared with placebo. More importantly, terlipressin is the only pharmacologic agent that significantly reduces mortality compared with placebo [37]. Somatostatin has been used in the pharmacological treatment of variceal hemorrhaging because it leads to splanchnic vasoconstriction and decreases portal pressure without the adverse effects of vasopressin on the systemic circulation. Some side effects such as nausea, vomiting, and hyperglycemia may occur in up to one-third of patients. Several randomized controlled studies have shown its efficacy in controlling bleeding [38–40]. However, somatostatin did not reduce mortality [11]. Octreotide and vapreotide are cyclic synthetic somatostatin analogs with longer half-lives than native somatostatin. Octreotide is safe and few major side effects are reported. The efficacy of octreotide as a single therapy for variceal bleeding is controversial, because two studies using octreotide or placebo after the control of the initial bleeding episode failed to show any difference in early rebleeding or mortality between the two treatment groups [41, 42]. Tachyphylaxis is another limitation [43]. However, octreotide appears to be useful as an adjunct to endoscopic therapy (endoscopic sclerotherapy or EVL) to achieve hemostasis [36].

The current management cornerstone of variceal hemorrhage is endoscopic treatment. Endoscopic therapy is capable of stopping bleeding in nearly 90% of patients. Endoscopy should be performed as soon as possible and not more than 12 hours after presentation. The two endoscopic methods available are sclerotherapy and variceal ligation. Endoscopic sclerotherapy arrests hemorrhage in 80% to 90% of patients and decreases the risk for early rebleeding, although an improvement in patient survival has never been shown [11]. EVL is a relatively newer therapeutic modality and has replaced endoscopic sclerotherapy as the endoscopic procedure of choice due to more effective control of bleeding, obliteration of varices in fewer treatment sessions, a lower rebleeding rate, and lower mortality [44]. Therefore, by consensus, EVL is the preferred form of endoscopic therapy [3, 10]. However, endoscopic sclerotherapy is an option when EVL is not available or technically difficult. 

Despite urgent endoscopic and/or pharmacologic therapy, variceal hemorrhages cannot be controlled or recured in about 10% to 20% of patients. In this case the patient should be offered an additional treatment before the patient's clinical status deteriorates. Transjugular intrahepatic portosystemic shunt (TIPS) has clinical efficacy as salvage therapy in whom standard combination therapy with endoscopic and pharmacological therapy fails, but the mortality rate is high (30–50%) because these patients usually have deteriorated liver function [23, 45, 46]. Both TIPS and surgical shunts are extremely effective in controlling variceal bleeding (control rate approaches 95%) but due to the efficacy, simplicity, and a better cost-effectiveness ratio of TIPS, surgical shunts have been practically abandoned [47]. 

The high mortality associated with the use of TIPS as a rescue treatment raises the question of whether patients with poor prognostic indicators might benefit from a more aggressive therapeutic approach. Two randomized, controlled trials have shown that an early placement of such a shunt (within up to 72 hours after admission) was associated with a reduction in failure to control bleeding, lower incidence of rebleeding, and a decreased mortality rate among high-risk patients (Child-Pugh C or an HVPG of >20 mmHg) [48, 49]. In addition, the TIPS group did not have an increased incidence of hepatic encephalopathy. Therefore, the early use of TIPS could be considered in these patients. 

The use of a Sengstaken-Blakemore or Minnesota tube can be a life-saving maneuver if medical and endoscopic measures fail to arrest the hemorrhage. Pneumatic compression of the fundus and the lower esophagus stops bleeding in approximately 85% of cases. Bleeding recurs after deflation in over half of the cases within 48 hours of placement and major complications including aspiration pneumonia and esophageal perforation may occur in up to 20% to 30% of patients. Balloon tamponade is only used as a temporary measure (inflated for 12 h or less) to control bleeding while a definitive therapy (TIPS or endoscopic therapy) is planned.

## 4. Prevention of Recurrent Variceal Hemorrhage (Secondary Prophylaxis)

Patients surviving in the first episode of variceal hemorrhages are at high-risk of recurrent bleeding, with a mortality of 33%, and thus should have secondary therapy to prevent further variceal bleeding [11]. The main means of secondary prophylaxis are pharmacological, by endoscopic treatment or a combination, or the use of shunts, usually a TIPS. 

All patients who are deemed compliant and have no contraindications should be considered for pharmacologic therapy. Nonselective *β*-blockers significantly decreased the risk of rebleeding and mortality [50]. Endoscopic sclerotherapy does significantly decrease rebleeding rates and mortality, but it has been associated with serious complications, the most common of which are esophageal stricture and bleeding from treatment-induced ulcers [51]. Endoscopic sclerotherapy has been replaced by EVL, since it has significantly better outcomes (regarding rebleeding, lower mortality, and complication) compared with sclerotherapy [11, 52]. The results of randomized, controlled trials comparing variceal ligation with *β*-blockers (nadolol) showed that combination treatment gives the lowest rebleeding rates, but without differences in survival [53]. The combination therapy of EVL and nonselective *β*-blockers for the prevention of recurrent variceal hemorrhaging is now the preferred therapy [6]. Patients who are intolerant or have contraindications to pharmacological therapy should receive EVL alone. 

Finally, TIPS in secondary prophylaxis has been shown to lower rebleeding rates when compared to the endoscopic and pharmacologic therapy [54]. However, no mortality benefit has been demonstrated with TIPS and its use is associated with higher costs and incidence of hepatic encephalopathy. Therefore, the use of TIPS in secondary prophylaxis is not recommended. However, TIPS or surgical shunt should be considered in patients with Child-Pugh A or B who experience recurrent variceal hemorrhaging despite combined treatment with EVL and nonselective *β*-blockers [55]. Liver transplantation should be considered for patients who meet indications for liver transplantation.
